# Three Cases from My Note Book

**Published:** 1855-10

**Authors:** S. H. Harvey

**Affiliations:** Washington, Ark.


### ARTICLE VIII.

*Three Cases from my Note Book.* By S. H. Harvey, M. D., Washington, Ark.

Messrs. Editors:—I send you from my *note book*, the three following cases: if you deem them of sufficient interest to justify a place in the Journal, you will lay them before the profession.

Case I.—The extraction of a superior right lateral incisor *through the nostril.*

The patient, a negro man about 35 years old, by accident, while chopping wood, a billet struck him on the mouth, and knocked entirely away, as he supposed, the right lateral incisor. Eight years after he was troubled by some hard substance making its appearance in his right nostril, and which finally resulted in the extraction of the missing tooth from that *orifice;* two years from the time he first discovered it, and ten from the date of the accident.

Case II.—The subject of this case was a young lady of sanguineous temperament, aged 15 years, and of scrofulous diathesis. She was brought to my office in May last, by her brother and family physician, (Dr. Jones,) to consult me in reference to a large cartilaginous tumor, situated immediately back and below the left ear. Had been growing some twelve months. No soreness to the touch, but very firm and hard. On examining her teeth, found, on the *same side*, the large left inferior molar in a very diseased condition, and eight other teeth, mostly on the same side, carious. The latter I filled, and extracted the former. The gums in the vicinity of this tooth were tinged and much inflamed. The tumor we decided not to touch for the present, believing that this large diseased tooth was the offending cause.

I saw this young lady a few days since, and to my great satisfaction found the tumor greatly diminished, at least two-thirds, and evidently fast disappearing, about three months from the date of extraction.

Case III.—This subject, also a young lady, aged 16, whose front teeth, in the superior maxillary, appeared in the following singular manner. On the right side, there was *no* lateral incisor, while on the left there were *two* about the same size and shape, and the cuspidatus of this side was immediately over the first bicuspis, and completely beyond or outside the dental arch, so as to destroy, in a great measure, the natural appearance of her mouth. This tooth I extracted, and it measured *one inch and a quarter in length!*

Washington, Ark., 21*st August*, 1855.

